# Optimal planning target margin for prostate radiotherapy based on interfractional and intrafractional variability assessment during 1.5T MRI-guided radiotherapy

**DOI:** 10.3389/fonc.2023.1337626

**Published:** 2023-12-20

**Authors:** Jina Kim, Jiwon Sung, Seo Jin Lee, Kang Su Cho, Byung Ha Chung, Dongjoon Yang, Jihun Kim, Jun Won Kim

**Affiliations:** ^1^ Department of Radiation Oncology, Gangnam Severance Hospital, Yonsei University College of Medicine, Seoul, Republic of Korea; ^2^ Department of Urology, Prostate Cancer Center, Gangnam Severance Hospital, Yonsei University College of Medicine, Seoul, Republic of Korea

**Keywords:** prostate cancer, PTV margin, MRI-guided radiotherapy, interfractional setup margin, intrafractional motion, interobserver variability

## Abstract

**Introduction:**

We analyzed daily pre-treatment- (PRE) and real-time motion monitoring- (MM) MRI scans of patients receiving definitive prostate radiotherapy (RT) with 1.5 T MRI guidance to assess interfractional and intrafractional variability of the prostate and suggest optimal planning target volume (PTV) margin.

**Materials and methods:**

Rigid registration between PRE-MRI and planning CT images based on the pelvic bone and prostate anatomy were performed. Interfractional setup margin (SM) and interobserver variability (IO) were assessed by comparing the centroid values of prostate contours delineated on PRE-MRIs. MM-MRIs were used for internal margin (IM) assessment, and PTV margin was calculated using the van Herk formula.

**Results:**

We delineated 400 prostate contours on PRE-MRI images. SM was 0.57 ± 0.42, 2.45 ± 1.98, and 2.28 ± 2.08 mm in the left-right (LR), anterior-posterior (AP), and superior-inferior (SI) directions, respectively, after bone localization and 0.76 ± 0.57, 1.89 ± 1.60, and 2.02 ± 1.79 mm in the LR, AP, and SI directions, respectively, after prostate localization. IO was 1.06 ± 0.58, 2.32 ± 1.08, and 3.30 ± 1.85 mm in the LR, AP, and SI directions, respectively, after bone localization and 1.11 ± 0.55, 2.13 ± 1.07, and 3.53 ± 1.65 mm in the LR, AP, and SI directions, respectively, after prostate localization. Average IM was 2.12 ± 0.86, 2.24 ± 1.07, and 2.84 ± 0.88 mm in the LR, AP, and SI directions, respectively. Calculated PTV margin was 2.21, 5.16, and 5.40 mm in the LR, AP, and SI directions, respectively.

**Conclusions:**

Movements in the SI direction were the largest source of variability in definitive prostate RT, and interobserver variability was a non-negligible source of margin. The optimal PTV margin should also consider the internal margin.

## Introduction

Definitive radiotherapy (RT) is a vital multimodal treatment option for prostate cancer, the most common malignant tumor in men (1, 2). Definitive RT demonstrates excellent local control rates from 85–90%, and randomized clinical trials have indicated that dose escalation further improves the local control rate (3–6). Consequently, increasing focus has been laid on hypofractionated RT or stereotactic body radiotherapy (SBRT) to treat prostate cancer (7). However, the risk of treatment-related toxicity increases with dose escalation and hypofractionation (8, 9).

A higher RT dose is delivered at each fraction in hypofractionated RT or SBRT; therefore, accurate and precise target localization is necessary. Minor deviations may lead to undercoverage of target volumes and higher toxicity rates (10). Several studies have attempted to assess the optimal target margin in prostate SBRT using image-guided RT (IGRT); however, they obtained limited information on prostate movement owing to the lack of full-time continuous monitoring or the use of X-ray-based image guidance (11, 12).

MRI-guided radiotherapy (MRIgRT) facilitates improved soft tissue contrast and better visualization of the target and surrounding tissues compared with that using X-ray-based IGRT. The most recent versions of MRIgRT systems, such as the ViewRay and Elekta MR-LINAC systems, combine intensity-modulated radiotherapy (IMRT) and real-time MRI-guidance with motion-monitoring capability, which allows online adaptive planning (ART) (13–15). MRIgRT with online adaptation for the prostate is expected to better address the daily changes of the target and organs at risk (OAR), such as prostate swelling, bladder filling, or rectal gas movement, and provide improved tumor control and reduced toxicities (16).

Furthermore, target volume can be reduced by accurately assessing the inter- and intrafractional variabilities. Previous studies have reported a significant reduction of the planning target volume (PTV) with adaptive planning using MRIgRT (17, 18). Interobserver variability is another important source of uncertainty in fractionated radiotherapy with online adaptation. Concerns for interobserver variability arise when recontouring the target and OARs for ART, even with advanced imaging techniques (19).

Accordingly, we aimed to assess interfractional and intrafractional variability during 1.5 T MRIgRT for the prostate and suggest the optimal PTV margin for definitive prostate RT. We delineated 400 prostate contours on daily pretreatment MRI images for analysis and presented the results of the comprehensive analysis.

## Materials and methods

### Study population

Patients with prostate cancer treated with MRIgRT between August 2021 and July 2022 were screened for inclusion in this study. Patient inclusion criteria were as follows: a) age ≥20 years at the time of treatment, b) histologically confirmed prostate cancer, and c) treated with definitive radiotherapy. One patient was excluded owing to extensive urinary bladder invasion of the primary tumor. This study was approved by the institutional review board (IRB) of our institution (IRB approval no. 3-2022-0413), and the requirement for informed consent was waived owing to the retrospective study design.

### MRIgRT workflow

Planning CT was performed for every patient, with 2 hours of bladder filling and an empty rectum. The clinical target volume (CTV) encompassed the whole prostate, and pelvic lymph nodes (presacral, external iliac, internal iliac, obturator) were selectively included for patients at high risk. The PTV was obtained by expanding the CTV with 5 mm margins in all directions. An RT dose of 60 Gy in 20 fractions was prescribed for the prostate and 44 Gy in 20 fractions for the pelvic lymph nodes, with simultaneous integrated boosts. All 20 fractions were delivered with 1.5 T MR-Linac (Unity, Elekta AB, Stockholm, Sweden).

**Figure 1:**
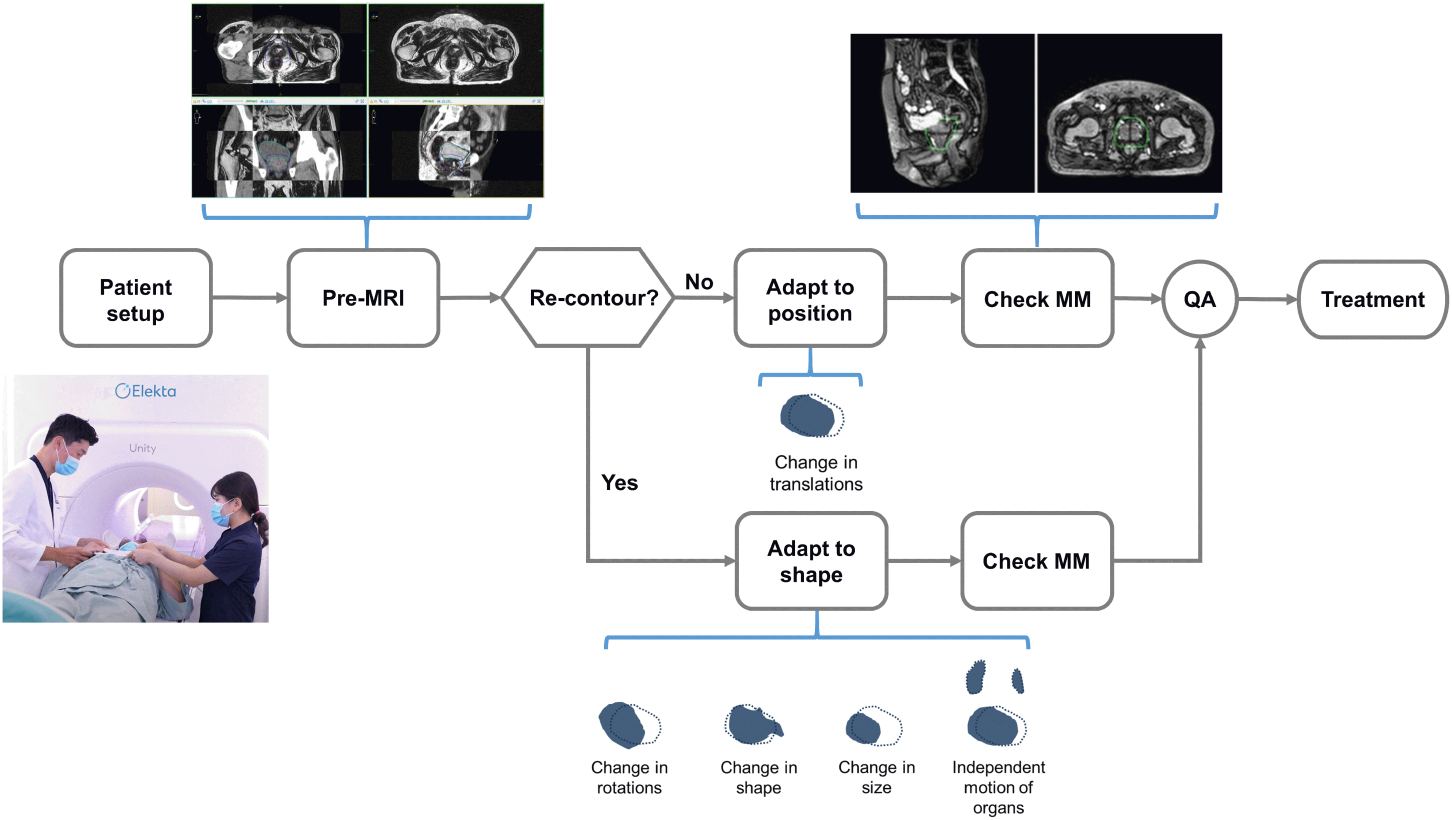
Workflow of MR-guided radiotherapy.

T2-weighted three-dimensional pre-treatment MRI (PRE) was acquired at each treatment, contours from the planning CT were automatically propagated and manually adapted to the PRE-MRI, and plan re-optimization was initiated in the Monaco MR-Linac treatment planning system. The adapt-to-position (ATP) algorithm was selected if only translational movement was required. The adapt-to-shape (ATS) algorithm was selected when changes in the shape or size of the target and OAR were observed. Details of the workflow have been described elsewhere (13). When necessary, a radiation oncologist performed re-contouring, which was then followed by an online adaptive planning and an online quality assurance process. Motion monitoring- (MM) MRI images were acquired with the 3D balanced turbo field echo sequence immediately before and during radiation delivery to monitor the intrafractional movement of the prostate and OARs. The workflow of MRIgRT is presented in **Figure 1**.

### Data analysis for planning target margin

We collected simulation CT images, PRE-MRIs, and MM-MRIs of the 20 patients to determine the optimal planning target margin for prostate cancer. PRE images for the first five fractions of the 20 patients were collected. We performed rigid registration between PRE-MRI and planning CT images based on pelvic bony and prostate anatomy using the MIM software (MIM Software Inc., Cleveland, OH, USA) and produced X-ray-based IGRT and MRI-guided ART processes, respectively. A radiation oncologist (JK) delineated only the prostate gland on all the PRE-MRIs and determined the centroid values, which were compared between the PRE and planning CT images to determine the interfractional setup margin (SM). Another radiation oncologist (JWK), a physicist (JWS), and a radiotherapist (DJY) delineated the prostate gland, and the differences in prostate centroid values between each of the four observers were compared to determine interobserver variability (IO). For internal margin (IM) assessment, two-dimensional cine MRI images of the five patients who had all three sets of coronal, sagittal, and axial cine images were acquired. The images were initially saved as binary files and were converted to a metadata format using an in-house software tool. Subsequently, the images were superimposed with a grid of 1 mm spacing, and the maximum contour displacement was determined in the left-right (LR), anterior-posterior (AP), and superior-inferior (SI) directions. A cine image of a patient with a superimposed 1-mm grid is presented in **Supplementary Figure 1.**

We used the van Herk formula to calculate the appropriate PTV margin required for definitive RT of the prostate, a formula which incorporates both the systematic errors (Σ) and random errors (σ) of SM, IM, and IO (20, 21). The systematic error was obtained by the standard deviation of the mean, and random error was obtained by the root mean square of the standard deviations. The appropriate PTV margins in each of the LR, AP, and SI directions were assessed using the formula below:


$$\text{PTV margin}=2.5\sqrt{\Sigma_{SM}^{\;2}+\Sigma_{IM}^{\;2}+\Sigma_{IO}^{\;2}}+0.7\sqrt{\delta_{SM}^{\;2}+\delta_{IM}^{\;2}+\delta_{IO}^{\;2}}$$

## Results

Patient and tumor characteristics are presented in **Table 1**. The median age was 77 years (interquartile range [IQR], 72–83), and 11 patients (55%) had stage T2 cancer. Regarding risk classification, one patient (5%) was low risk, five (25%) were unfavorable intermediate risk, four (20%) were high risk, and 10 (50%) were very high risk. The median volumes of the prostate, bladder, and rectum were 42.5, 182.6, and 62.8 mL, respectively. Regarding the radiation field, six patients (30%) received RT only to the prostate, two (10%) received RT to the prostate and the seminal vesicles, and 12 (60%) received RT to the prostate and pelvic lymph node area. All patients completed their RT course. Nineteen patients (95%) received hormone therapy concurrently with RT, and 14 (70%) continued to receive hormone therapy after RT.

**Table 1:** Patient and treatment characteristics.

Variables	N/Median	%/IQR
Age	77	72.3-82.8
Initial PSA (ng/mL)	10.3	6.4-83.6
T stage		
2a	2	10
2b	4	20
2c	5	25
3a	0	0
3b	6	30
4	3	15
Gleason score		
3 + 3	1	5
3 + 4	4	20
4 + 3	3	15
4 + 4	6	30
4 + 5	5	25
5 + 4	1	5
Risk group		
Low risk	1	5
Favorable intermediate	0	0
Unfavorable intermediate	5	25
High risk	4	20
Very high risk	10	50
Prostate volume (cc)	42.5	28.9-47.2
Bladder volume (cc)	182.6	149.3-238.6
Rectum volume (cc)	62.8	49.9-70.6
Radiotherapy field		
Prostate only	6	30
Prostate + seminal vesicles	2	10
Whole pelvis	12	60
Prostate Radiotherapy dose		
3 Gy x 20 fx	20	100

IQR, interquartile range; PSA, prostate-specific antigen; fx, fractions.

A total of 100 PRE-MRIs were collected, and 400 prostate contours were delineated. Regarding intrafractional variability assessment, 25 MM-MRIs were analyzed.

### Interfractional variability (Setup margin)

**Figure 2:**
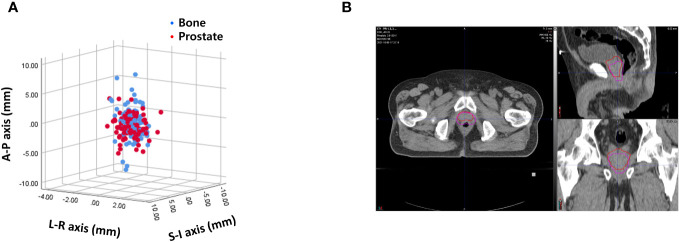
**(A)** Interfractional variability based on bone anatomy and prostate registration. **(B)** A case of maximum interfractional variability.

**Figure 3:**
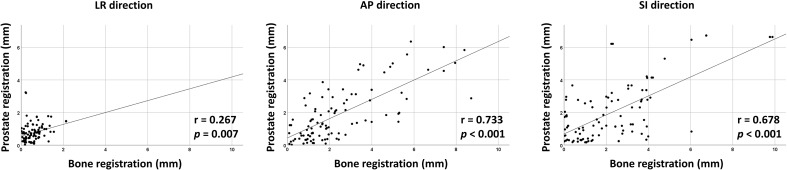
Correlation between bone- and prostate-based image registration in LR, AP, and SI directions.

Interfractional variability was measured by calculating the difference in prostate contours between the PRE-MRI and planning CT images using the bone and prostate localization methods. One hundred prostate contours delineated by a board-certified radiation oncologist (JK) were used for interfractional variability assessment. Interfractional variability expressed in mean ± standard deviation was 0.57 ± 0.42, 2.45 ± 1.98, and 2.28 ± 2.08 mm in the LR, AP, and SI directions, respectively, after bone localization, and 0.76 ± 0.57, 1.89 ± 1.60, and 2.02 ± 1.79 mm in the LR, AP, and SI directions, respectively, after prostate localization (**Figure 2A**). A case of maximum interfractional variability is displayed in **Figure 2B**. We analyzed the correlation between bone anatomy and prostate registration and observed a strong correlation in the AP direction, with a Pearson r value of 0.733 (*p*< 0.001), and a moderate correlation in the SI direction, with a Pearson r value of 0.678 (*p*< 0.001). The Pearson r value in the LR direction was 0.267 (*p* = 0.007) (**Figure 3**).

### Interobserver variability

**Figure 4:**
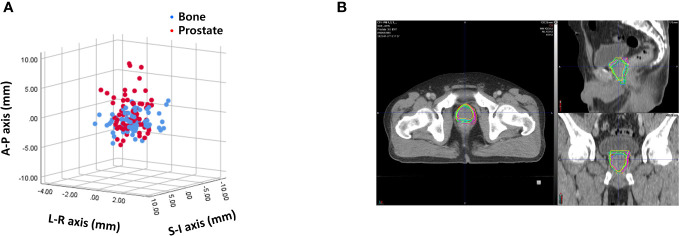
**(A)** Interobserver variability based on bone anatomy and prostate registration. **(B)** A case of maximum interobserver variability.

Interobserver variability was measured by calculating the difference of 400 prostate contours on each PRE-MRI by four observers using the bone and prostate localization methods. Interobserver variability expressed in mean ± standard deviation was 1.06 ± 0.58, 2.32 ± 1.08, and 3.30 ± 1.85 mm in the LR, AP, and SI directions, respectively, after bone localization and 1.11 ± 0.55, 2.13 ± 1.07, and 3.53 ± 1.65 mm in the LR, AP, SI directions, respectively, after prostate localization (**Figure 4A**). The average and standard deviation values of target centroid displacement by each observer are listed **in Supplementary Tables 1, 2**. A case of maximum interobserver variability is displayed in **Figure 4B**.

### Intrafractional variability (Internal margin)

Intrafractional variability was assessed by measuring the maximum displacement of the prostate on the MM images. The resulting values were 2.12 ± 0.86, 2.24 ± 1.07, and 2.84 ± 0.88 mm in the LR, AP, and SI directions, respectively.

### PTV margin calculation

The systematic (Σ) and random error (σ) values of SM, IM, and IO are listed in **Table 2**. The PTV margin calculated using the van Herk formula was 2.21, 5.16, and 5.40 mm in the LR, AP, and SI directions, respectively.

**Table 2:** Systematic and random errors of setup margin (SM), internal margin (IM), and interobserver variability (IO).

		L-R	A-P	S-I
**SM**	Σ	0.23	1.20	0.95
	σ	0.35	1.09	0.85
**IM**	Σ	0.33	0.81	1.41
	σ	0.45	0.71	1.04
**IO**	Σ	0.48	0.78	0.23
	σ	0.72	0.73	0.85
**PTV margin (mm)**		2.21	5.16	5.40

L-R, left-right; A-P, anterior-posterior; S-I, superior-inferior; SM, setup margin; IM, internal margin; IO, interobserver variability; PTV, planning target volume.

### PTV margin for potential SBRT cases

Data of 11 patients with clinical T1-2, N0 stage cases were selected and analyzed to suggest the optimal PTV margin for potential SBRT cases. Interfractional variability was 0.57 ± 0.48, 2.04 ± 1.55, and 2.60 ± 2.44 mm in the LR, AP, and SI directions, respectively, after bone localization and 0.60 ± 0.45, 1.73 ± 1.51 mm, and 1.85 ± 1.92 mm in the LR, AP, and SI directions, respectively, after prostate localization. Interobserver variability was 1.17 ± 0.59, 2.42 ± 1.08, and 3.50 ± 1.76 mm in the LR, AP, and SI directions, respectively, after bone localization and 1.07 ± 0.50, 2.26 ± 1.30, and 3.46 ± 1.66 mm in the LR, AP, and SI directions, respectively, after prostate localization. Intrafractional variability was 2.07 ± 0.77, 2.33 ± 1.14, and 2.93 ± 1.00 mm in the LR, AP, and SI directions, respectively. The PTV margin calculated using the van Herk formula was 1.95, 4.58, and 5.35 mm in the LR, AP, and SI directions, respectively. The systematic (Σ) and random error (σ) values of SM, IM, and IO are listed in **Supplementary Table 3.**

## Discussion

In this study, we used daily pretreatment MRI images of 20 patients receiving RT to evaluate the interfractional and intrafractional variation of the prostate. The variation in the SI direction was the largest in SM, IM, and IO, and that in the LR direction was the smallest. The PTV margin is a determining factor for local control and toxicity in definitive prostate RT; therefore, an accurate assessment of the PTV margin is necessary, particularly with the implementation of hypofractionation and SBRT (22–24).

In our study, we observed that the interfractional and intrafractional variability of the prostate was greater in the SI and AP directions compared with that in the LR direction. This finding is in parallel with many others. A study by M.D. Anderson Cancer Center quantified the prostate and seminal vesicle interfractional movement by contouring the target and OARs on 369 CT scans and observed that the dominant variation was in the AP and SI directions (25). Huang et al. measured the intrafractional prostate motion of 20 patients with prostate cancer and observed that intrafractional movement predominantly occurred in the anterior and superior directions (26). Mah et al. also measured intrafractional prostate motion with cine-MRIs and observed that prostate movement mostly occurred anteriorly and superiorly (27). Wong et al. reviewed CT images of 329 patients with prostate cancer receiving image-guided RT to analyze the interfractional prostate shifts and reported that the shift in the AP direction was higher than that in the LR or SI directions (28).

Interfractional variability based on bone anatomy and prostate registration revealed a strong correlation in the AP direction. In contrast, the correlation was moderate in the SI direction and weak in the LR direction. Our finding differs from those of a previous Mayo Clinic study in which Beltran et al. observed a strong correlation in the LR direction (Pearson r = 0.89), a moderate correlation in the AP direction (Pearson r = 0.59), and a weak correlation in the SI direction (Pearson r = 0.35) (29). However, the method of prostate-based registration by these authors differed from ours: we used the outer contours of the prostate, whereas Beltran et al. used intraprostatic gold seeds, which may have resulted in such a difference. Nonetheless, the two studies are concordant in that bone registration cannot be a perfect surrogate for prostate registration; therefore, ART is necessary for accurate treatment delivery.

Interobserver variability was also a substantial source of variation in prostate RT. Previous studies also highlighted the clinical significance of interobserver variability. In a study from Princess Margaret Hospital, Toronto, Canada, five genitourinary oncologists analyzed prostate contours on five cone beam CT images and identified considerable disagreement (30). Variability decreased with improved soft tissue contrast imaging. Lütgendorf-Caucig et al. reported interobserver variability of the prostate target volume delineation and observed larger variability for cone beam CT-based contouring than that for CT and MRI (31). Villeirs et al. compared the interobserver variation of prostate and seminal vesicle contours using CT alone and CT plus MRI and observed that CT plus MRI reduced CTV volume and the standard deviation values (32). Nevertheless, our findings revealed that interobserver variability should still be accounted for when assessing the prostate PTV margin. In our study, the observers did not discuss how to contour the prostate prior to contouring. One way to reduce interobserver variability may be to use the same consensus guideline (33).

In this study, we assessed SM, IM, and IO and suggested a PTV margin of 2.2, 5.2, and 5.4 mm in the LR, AP, and SI directions, respectively. Beltran et al. compared four localization methods of skin marks using tattoos, pelvic bony anatomy, and intraprostatic gold seeds using a 5 mm action threshold and no threshold and suggested that localization using gold fiducials can largely reduce the PTV margin compared to skin marks or bony anatomy for prostate RT. The suggested PTV margin when using intraprostatic gold seeds was 4.8, 5.2, and 5.4 mm in LR, AP, and SI directions, respectively, which was twice larger than our result in the LR direction but identical to our results in the AP and SI directions (29). MRIgRT may be preferred for patient comfort and safety, considering that fiducial insertion is invasive and carries the risk of infection, albeit low (34, 35). We also assessed the PTV margin for patients with clinical T1-2, N0 disease, which are potential candidates for prostate SBRT, and observed that a smaller margin was necessary for these cases. This implies that smaller margins can be employed for selected SBRT cases; however, further validation with a larger patient cohort is necessary. Moreover, the PTV margin calculated using the van Herk formula based on the systematic (Ʃ) and random (σ) error values obtained in this study can be further reduced to 1.4, 4.5, and 5.2 mm in the LR, AP, and SI directions, respectively, when IO was neglected. Recently, hydrogel rectal spacer insertion has been attempted to further reduce PTV margin for prostate SBRT. Cuccia et al. analyzed the translational and rotational shifts of patients who underwent MRI-guided prostate SBRT with or without hydrogel spacer, and observed significantly minimized rotational shifts in the antero-posterior axis with hydrogel spacer (36). Similarly, Mazzola et al. analyzed the impact of hydrogel spacer on seminal vesicle motion in patients undergoing MRI-guided prostate SBRT and found significant reduction of translational shift in the cranio-caudal axis with hydrogel spacer (37). While the method of intrafractional variability assessment of the previously mentioned studies differed from the one used in this study—the former using pre- and post-treatment MRI images and the latter using real time MM images—all highlight the importance of PTV margin minimization and appropriate patient selection for prostate SBRT.

The strengths of this study include the implementation of MRIgRT, a non-invasive method with superior soft tissue contrast, and the collection of daily MRIs for image guidance and real-time motion monitoring to assess all potential sources of variability during RT (SM, IM, and IO) and suggest an optimal PTV margin using a well-known formula. However, this study also had some limitations, mainly attributed to its retrospective design. Image registration was performed by one radiation oncologist, which may have introduced bias. Patients with various risk types and RT fields were included, giving rise to a heterogeneous population. However, 400 prostate contours were delineated and analyzed together in this study, strengthening statistical power. Moreover, all three sets of axial, coronal, and sagittal images of each patient were used for analysis, allowing an unimpaired assessment.

In conclusion, movements in the SI direction were a major source of variability in definitive prostate RT. Moreover, interobserver variability was a non-negligible source of margin. The optimal PTV margin should also consider the internal margin, particularly in the SI direction. Further studies with dosimetric evaluation should be performed to better assess the PTV margin for definitive prostate RT.

## Data availability statement

The raw data supporting the conclusions of this article will be made available by the authors, without undue reservation.

## Ethics statement

The studies involving humans were approved by Gangnam Severance Hospital institutional review board. The studies were conducted in accordance with the local legislation and institutional requirements. Informed consent was waived due to the retrospective study design.

## Author contributions

JK: Data curation, Formal analysis, Methodology, Visualization, Writing – original draft, Writing – review & editing. JS: Data curation, Methodology, Writing – review & editing. SJL: Data curation, Writing – review & editing. KSC: Data curation, Writing – review & editing. BHC: Data curation, Writing – review & editing. DY: Data curation, Writing – review & editing. JHK: Methodology, Writing – review & editing. JWK: Conceptualization, Funding acquisition, Project administration, Supervision, Writing – review & editing.

## Supplementary material

Supplementary Figure 1 Exemplary axial, coronal, and sagittal image of a cine MRI image superimposed with a grid of 1mm spacing. Thick and thin lines indicate 1 cm and 1 mm spacing, respectively.
