# Supplementary figures and images for: Optimal planning target margin for prostate radiotherapy based on interfractional and intrafractional variability assessment during 1.5T MRI-guided radiotherapy
Source: Front Oncol. 2023 Dec 20;13:1337626. doi: 10.3389/fonc.2023.1337626 (PMC10761547; doi:10.3389/fonc.2023.1337626)

## Slide 1
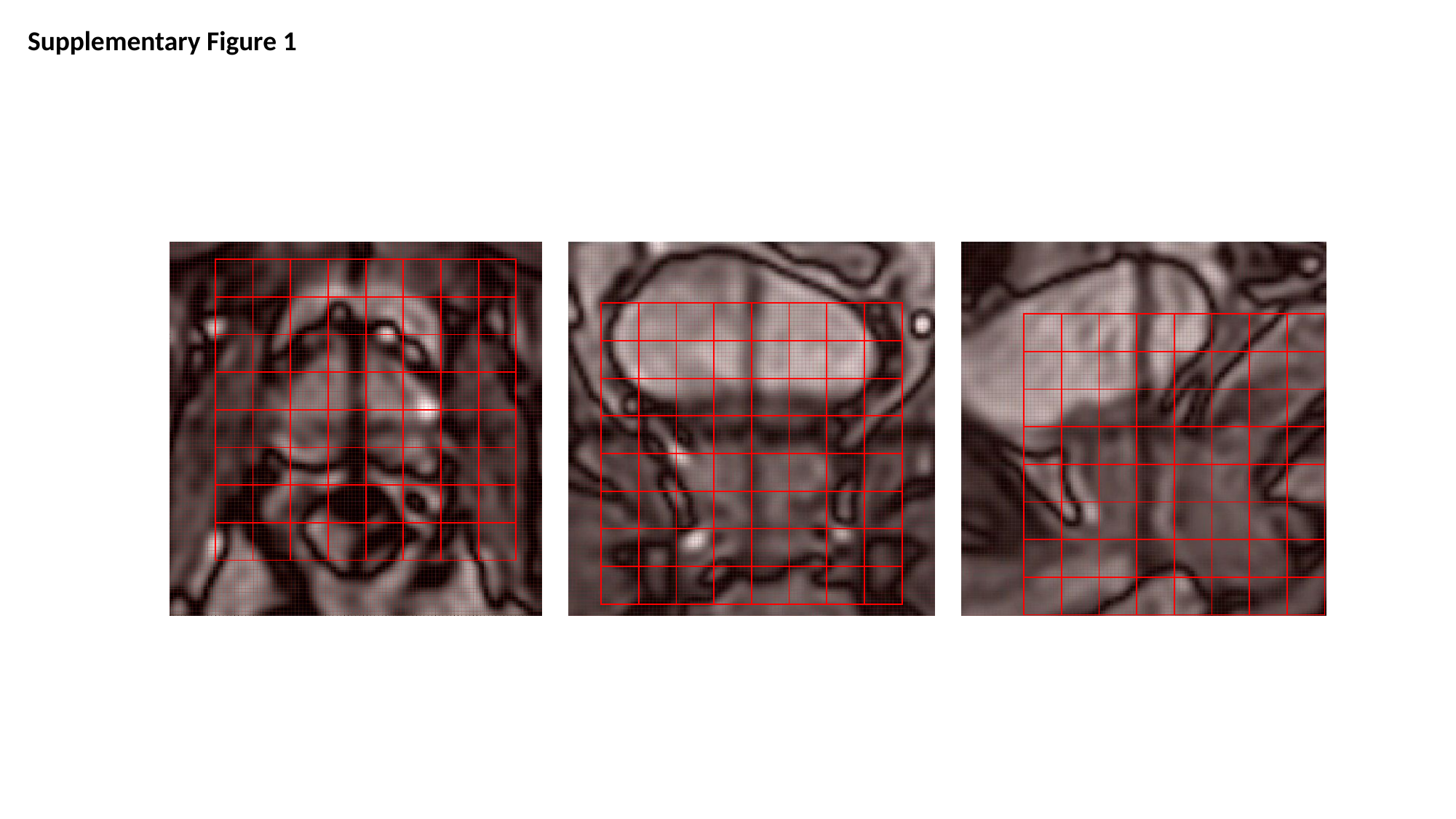

Supplementary Figure 1
| | | | | | | | |
| --- | --- | --- | --- | --- | --- | --- | --- |
| | | | | | | | |
| | | | | | | | |
| | | | | | | | |
| | | | | | | | |
| | | | | | | | |
| | | | | | | | |
| | | | | | | | |
| | | | | | | | |
| --- | --- | --- | --- | --- | --- | --- | --- |
| | | | | | | | |
| | | | | | | | |
| | | | | | | | |
| | | | | | | | |
| | | | | | | | |
| | | | | | | | |
| | | | | | | | |
| | | | | | | | |
| --- | --- | --- | --- | --- | --- | --- | --- |
| | | | | | | | |
| | | | | | | | |
| | | | | | | | |
| | | | | | | | |
| | | | | | | | |
| | | | | | | | |
| | | | | | | | |

Supplement: Supplementary Figure 1 — Exemplary axial, coronal, and sagittal image of a cine MRI image superimposed with a grid of 1mm spacing. Thick and thin lines indicate 1 cm and 1 mm spacing, respectively. [file Presentation_1.pptx]
