# Supplementary material for: Optimal planning target margin for prostate radiotherapy based on interfractional and intrafractional variability assessment during 1.5T MRI-guided radiotherapy
Source: Front Oncol. 2023 Dec 20;13:1337626. doi: 10.3389/fonc.2023.1337626 (PMC10761547; doi:10.3389/fonc.2023.1337626)
Supplement: Supplementary file 2 [file Table_1.docx]

Supplementary Table 1. Average and standard deviation values of target centroid displacement by each observer on bone localization

| **Observer** |  | **L-R** | **A-P** | **S-I** |
| --- | --- | --- | --- | --- |
| **A** | Average (mm) | 0.57 | 2.45 | 2.28 |
|  | Standard deviation (mm) | 0.42 | 1.98 | 2.08 |
| **B** | Average (mm) | 0.42 | 2.02 | 1.25 |
|  | Standard deviation (mm) | 0.35 | 2.03 | 0.86 |
| **C** | Average (mm) | 0.46 | 2.48 | 1.68 |
|  | Standard deviation (mm) | 0.58 | 2.22 | 2.18 |
| **D** | Average (mm) | 0.55 | 2.14 | 0.92 |
|  | Standard deviation (mm) | 0.52 | 1.45 | 1.51 |

*Abbreviations: L-R, left-right; A-P, anterior-posterior; S-I; superior-inferior

Supplementary Table 2. Average and standard deviation values of target centroid displacement by each observer on prostate localization

| **Observer** |  | **L-R** | **A-P** | **S-I** |
| --- | --- | --- | --- | --- |
| **A** | Average (mm) | 0.76 | 1.89 | 2.02 |
|  | Standard deviation (mm) | 0.57 | 1.6 | 1.79 |
| **B** | Average (mm) | 0.47 | 1.39 | 0.89 |
|  | Standard deviation (mm) | 0.53 | 1.34 | 0.66 |
| **C** | Average (mm) | 0.63 | 1.79 | 2.06 |
|  | Standard deviation (mm) | 0.66 | 1.53 | 2.19 |
| **D** | Average (mm) | 0.81 | 1.92 | 1.98 |
|  | Standard deviation (mm) | 0.71 | 1.22 | 1.84 |

*Abbreviations: L-R, left-right; A-P, anterior-posterior; S-I; superior-inferior

Supplementary Table 3. Systematic and random errors of setup margin (SM), internal margin (IM), and interobserver variability (IO) for T1-2, N0 patients

|  |  | **L-R** | **A-P** | **S-I** |
| --- | --- | --- | --- | --- |
| **SM** | Σ | 0.19 | 0.67 | 0.93 |
|  | σ | 0.28 | 0.98 | 0.76 |
| **IM** | Σ | 0.34 | 0.96 | 1.42 |
|  | σ | 0.44 | 0.71 | 0.95 |
| **IO** | Σ | 0.38 | 0.82 | 0.09 |
|  | σ | 0.67 | 0.78 | 0.99 |
| **PTV margin (mm)** |  | 1.95 | 4.58 | 5.35 |
|  |  |  |  |  |

*Abbreviations: L-R, left-right; A-P, anterior-posterior; S-I; superior-inferior; SM, setup margin; IM, internal margin; IO, interobserver variability; PTV, planning target volume
